# Supplementary material for: WordCommentsAnalyzer: A windows software tool for qualitative research
Source: F1000Res. 2018 Sep 4;7:536. Originally published 2018 May 3. [Version 2] doi: 10.12688/f1000research.14819.2 (PMC6137411; doi:10.12688/f1000research.14819.2)
Supplement: Supplementary file 3 [file f1000research-7-17614-s0002.tgz › b2a79496-4ada-47c4-9682-a99ce867836d.zip!/Eight Iranian Health Journals/Yafteh _44.docx]

© Yafteh  . This work is licensed under CC BY 4.0 (https://creativecommons.org/licenses/by/4.0/).

Volume & Issue: دوره 10 - شماره 1

Title: بررسی اپیدمیولوژیک موارد مسمومیت با سموم حشره کش (ارگانوفسفره و ارگانوکلره) در بیماران بستری شده در بیمارستان شهدای عشایر خرم آباد در شش ماه اول سال 1385

http://yafte.lums.ac.ir/article-1-63-fa.html

مقدمه: مسمومیت یکی از فوریتهای شایع پزشکی است. هر ساله تعداد زیادی بر اثر مسمومیت های شیمیایی و دارویی به اورژانس ها مراجعه و تحت درمان قرار می گیرند. تعدادی نیز به دنبال شدت عوارض حاصله فوت می شوند. هر چند در کشور های پیشرفته مسمومیت نیز، یک مشکل می باشد. ولی بیشتر مراجعین بیمارانی هستند که به قصد خودکشی اقدام به این کار می کنند. این مطالعه به منظور تعیین میزان شیوع مسمومیت با حشره کش های (ارگانوفسفره و ارگانوکلره) در مراجعین بستری شده در بیمارستان شهدای عشایر خرم آباد انجام گردید . مواد و روش‌ها: برای جمع آوری اطلاعات مربوط به همه مسموم شدگان با سموم حشره کش (ارگانوفسفره و ارگانوکلره) مراجعه کننده به بیمارستان شهدای عشایر خرم آباد در شش ماه اول سال 1385 پرسشنامه هایی طراحی و تکمیل گردید . یافته‌ها: از بین 153 بیمار مسموم مراجعه کننده، 1/77% با سموم ارگانوفسفره و 9/22% با سموم ارگانوکلره دچار مسمومیت شده بودند. 6/34% بیماران در گروه سنی 21-17 سال بودند. 5/57% مسمومین زن و 5/42% آنها مرد بودند. سواد اغلب بیماران در سطح راهنمایی و پایینتر بود. 1/64% از آنها مجرد بودند. 5/91% مسمومیت ها از نوع اقدام به خودکشی بود. در مجموع 12 نفر از بیماران درگذشتند که 7 نفر در اثر مسمومیت با سموم ارگانوکلره و به علت تشنج مقاوم به درمان و 5 نفر در اثر سموم ارگانوفسفره صورت گرفت. اغلب مرگ ها در اثر عوارض تنفسی مانند ARDS و پنومونی آسپیراسیون رخ داد . بحث و نتیجه‌گیری: یافته ها بیان می کند که باید به دلیل شیوع زیاد مسمومیت و مرگ و میر بالای آن با حشره کش ها، این موضوع مورد توجه جدی قرار بگیرد. همچنین آموزش دقیق کارکنان مراکز درمانی و برخورد صحیح با این مسمومیتها از اهمیت ویژه ای برخوردار است .

Title: مقایسه تاثیر اینداکشن به دو روش ضربانی و مداوم بر نتایج حاملگی در زنان باردار مراجعه کننده به بیمارستان 29 بهمن تبریز در سال 86- 1385

http://yafte.lums.ac.ir/article-1-64-fa.html

مقدمه: فعالیت ناکافی رحم بعنوان یک علت شایع و قابل اصلاح پیشرفت غیرطبیعی زایمان می­باشد که با تجویز اکسی­ توسین تصحیح می شود. این واقعیت که طولانی شدن بی ­مورد زایمان، ممکن است در بروز عوارض و مرگ ­و ­میر دوره پیش از تولد نقش داشته باشد، استفاده از تزریق داخل وریدی اکسی­توسین در درمان انواع مشخص انقباضات غیر مؤثر را باعث شده است . مواد و روش‌ها: در این مطالعه 100 زن باردار مراجعه­کننده به بیمارستان 29 بهمن تبریز در مطا لعه­ای نیمه­ تجربی شرکت­ کردند. به طوریکه بصورت تصادفی در گروه تحریک زایمان به روش ضربانی یا مداوم قرار ­گرفتند و پیشرفت زایمان ­ تحریک بیش از حد رحم، آپگار و هیپر ­ بیلی روبینمی نوزاد، مدت زمان زایمان و طول مرحله سوم زایمان اندازه­گیری شد . یافته‌ها: بر اساس یافته­های پژوهش، ازنظر پیشرفت زایمان، هیپراستیمولیشن در طی اینداکشن، آپگار و میزان هیپر بیلی روبینمی نوزاد، تفاوت معنی­داری بین دو گروه مشاهده نشد (05/0 p> ). ولی از نظر مدت زایمان (022/0 p= ) و نوع زایمان (008/0 p= ) و دوز مصرفی اکسی­توسین تفاوت معنی­دار بود (0005/0 p< ). بحث و نتیجه‌گیری: اثر تزریق ضربانی اکسی­توسین روش آسان تر و فیزیولوژیک­ تر ­برای ایجاد انقباضات موثر رحمی بوده و طول ­مدت ­زایمان­ و میزان زایمان غیر طبیعی (زایمان با واکیوم و سزارین­)­ را کاهش می­دهد. همچنین میزان مصرف اکسی­توسین در این روش نسبت به روش مداوم به طور قابل­ملا­حظه کمتر است .

Title: بررسی وضعیت و ضرورت تدریس آموزش خطاهای پزشکی از دیدگاه پزشکان عمومی شاغل در استان لرستان در سال 1385

http://yafte.lums.ac.ir/article-1-65-fa.html

مقدمه: خطاهای پزشکی یکی از مشکلات عمده بهداشت و درمان در جهان است که سالانه سبب مرگ و میر و عوارض جسمی و روحی برای بیماران و سایر افراد خانواده و حتی جامعه می شود، توجه به این موضوع در کشورهای مختلف در حال افزایش است. راهکارهای مختلفی برای مقابله با خطاهای پزشکی انتخاب شده است که یکی از آنها آموزش در دوران تحصیل است. هدف از انجام این طرح بررسی وضعیت آموزش خطاهای پزشکی در طی دوره تحصیلی و همچنین ضرورت تدریس این موضوع بعنوان یکی از سرفصل های آموزشی، با استفاده از نظرات پزشکان عمومی فارغ التحصیل دانشگاههای مختلف کشور در استان لرستان است . مواد و روش‌ها: برای اجرای تحقیق، پرسشنامه ای طراحی گردید که همکاران طرح با مراجعه حضوری به محل کار و مطب شخصی 200 تن از پزشکان عمومی پرسشنامه ها را تحویل و پس از تکمیل پرسشنامه توسط پزشکان، آنها را جمع آوری نموده و پس از تکمیل کلیه پرسشنامه ها اطلاعات آنها استخراج و توسط نرم افزار کامپیوتری SPSS آنالیز گردید . یافته‌ها: 90% از پزشکان عمومی شرکت کننده در این طرح بیان نمودند که موضوع جداگانه ای تحت عنوان خطاهای پزشکی در دوران تحصیل تدریس نشده است، 64% در طول دوره تحصیل مرتکب خطاهای پزشکی شده که 5/47% آن خطا در تشخیص بیماری بوده است، 60% بعد از فراغت از تحصیل نیز مرتکب خطاهای پزشکی شده اند که 51% آن هم در تشخیص بیماری بوده است. 5/72% بیان نمودند که بعد از فراغت از تحصیل در خصوص خطاهای پزشکی آموزش ندیده اند. همچنین 5/88% پزشکان، تدریس پیشگیری از خطاهای پزشکی را ضروری دانسته اند که40% آنها معتقد بودند که این آموزشها باید در حیطه تشخیص بیماری باشد. بحث و نتیجه‌گیری: با توجه به بررسی های بعمل آمده و اهمیت موضوع خطاهای پزشکی، تدریس آن در طی دوران تحصیل بعنوان واحدی جداگانه برای دانشجویان پزشکی امری ضروری بوده و پزشکان عمومی شاغل هم نیازمند تداوم آموزشهای مربوط هستند.

Title: اثر عصاره آبی برگ سنجد بر درد در موش صحرایی نر

http://yafte.lums.ac.ir/article-1-66-fa.html

مقدمه: مهار درد از دیرباز مورد توجه دانشمندان علوم پزشکی بوده وهمواره سعی شده تا داروهای موثرتری که عوارض جانبی کمتری هم داشته باشند معرفی گردند. در طب سنتی ایران برای گیاه سنجد آثار ضد التهاب و ضد درد عنوان شده است. در این مطالعه اثر ضد دردی عصاره آبی برگ گیاه سنجد مورد بررسی قرار گرفت . مواد و روش‌ها: برای بررسی اثر تسکینی گیاه سنجد از 35 سر موش صحرایی نر استفاده شد. پس از تهیه عصاره آبی اثر ضد دردی تزریق صفاقی دوزهای 25، 50، 100 میلی گرم بر کیلوگرم وزن از این عصاره در مقایسه با داروی ضد درد غیر استروئیدی دیکلوفناک با دوز5 میلی گرم بر کیلوگرم ازطریق تست فرمالین مورد مطالعه قرار گرفت . یافته‌ها: عصاره برگ سنجد بصورت وابسته به دوز اثر تسکینی مناسبی برهر دو مرحله درد ناشی از تزریق فرمالین داشت و این اثر، از اثر ضد دردی داروی دیکلوفناک قویتر بود Ø بحث و نتیجه‌گیری: اثر ضد دردی گیاه به دو طریق مرکزی و محیطی اعمال می گردد. برگ سنجد حاوی مواد موثره ترپنوئید و فلاونوئید است و بخشی از اثر ضد دردی این گیاه ناشی از اثر ضد التهابی مواد فوق الذکر می باشد .

Title: مقایسه بی حس کنندگی ترامادول و لیدوکائین در اعمال جراحی کوچک تحت بی حسی موضعی به روش بلوک زیر جلدی

http://yafte.lums.ac.ir/article-1-67-fa.html

مقدمه: مطالعات جدید نشان داده که ترامادول علاوه بر اثرات ضد درد اثر بی حس کنندگی موضعی هم دارد. مطالعه حاضر با هدف بررسی اثرات بیحس کنندگی موضعی ترامادول در مقایسه با لیدوکائین طراحی شد این پدیده که ترامادول اثرات بی حس کنندگی و بی دردی در استفاده بصورت زیر جلدی ایجاد کند خود می تواند انقلاب جدیدی در فصل بی حس کننده های موضعی باشد. مواد و روش‌ها: این مطالعه بصورت کارآزمایی بالینی تصادفی دو سویه کور روی 70 بیمار 50-20 ساله ASA class I ,II کاندید اعمال جراحی کوچک تحت بلوک زیرجلدی انجام شد. گروه T با ترامادول 2mg/kg و گروه L با لیدوکائین mg/kg 1 بلوک زیرجلدی شدند. میانگین شدت درد زمان تزریق، برش جراحی و سپس هر 15 دقیقه در دقایق 15 و30 و45 و سپس هر 2 ساعت تا 6 ساعت بعد از عمل بر اساس VAS (Visual Analuge Scale) ثبت شد. در طی جراحی اگر بود mg/kg 5/0 از داروی مورد مطالعه مجدداً تزریق می شد. دربخش نیز در صورت استامینوفن mg325 به بیماران تجویز شد. اطلاعات با استفاده از روش تکرار مکرر و کای اسکوار انالیز گردید. یافته‌ها: بین دو گروه تفاوتی در متوسط نمره درد بر اساس VAS درمقاطع زمانی مختلف بررسی وجود نداشت (181/ 0 = p ) همچنین از نظر درجه واکنش موضعی پوستی و درجه خونریزی و دریافت داروی اضافی و میزان بروز استفراغ تفاوتی بین دو گروه مشاهده نشد. تهوع به طور بارزی در گروه ترامادول(8/22%) نسبت به گروه لیدوکائین (5%) بیشتر بود (002 / 0= p ) 1/17% در گروه L و 6/8% در گروه T به نیاز به دوز اضافی بیحس کننده در طی عمل داشتند (48/0= p ). میزان نیاز به استامینوفن بعد از عمل در گروه لیدوکائین بیشتر بود . بحث و نتیجه‌گیری: در جراحیهای کوچک و در استفاده زیرجلدی، ترامادول mg/kg 2 دارای اثرات بیحس کننده موضعی و ضددرد مشابه mg/kg 1 لیدوکائین است و می تواند به صورت جایگزین آن بکار رود و میزان مصرف مسکن بعد از عمل را در بیماران کاهش دهد.

Title: بررسی نقص های فانکشنال در ورزشکاران دارای سابقه اسپرین مچ پا

http://yafte.lums.ac.ir/article-1-68-fa.html

مقدمه: علی رغم اهمیت محدودیت های فانکشنال و نقص های اجرایی در ورزشکاران دارای سابقه اسپرین مچ پا مطالعات اندکی در این زمینه انجام شده است. پژوهش حاضر به منظور بررسی نقص های فانکشنال در ورزشکاران دارای سابقه اسپرین مچ پا انجام گرفت. مواد و روش‌ها: آزمودنی های تحقیق شامل 40 ورزشکار حرفه ای بودند که به روش نمونه گیری تصادفی از بین بازیکنان تیم های استانی رشته های فوتبال، هندبال، بسکتبال و والیبال استان لرستان جمع آوری شدند. آزمودنی ها به دو گروه آسیب دیده (26 ورزشکار دارای سابقه اسپرین مچ پا) و گروه سالم (14 ورزشکار بدون سابقه اسپرین مچ پا) تقسیم شدند. در این تحقیق توصیفی که به صورت میدانی اجرا شد برای شناسایی نقص های فانکشنال از آزمون های عملکردی جهش به شکل هشت لاتین و جهش جانبی استفاده شد. همه آزمودنی ها در آزمون جهش به شکل هشت لاتین که شامل لی لی کردن در مسیری به طول 5 متر و به شکل هشت لاتین و جهش جانبی که شامل 10 جهش جانبی رفت و برگشت به طول 30 سانتیمتر بود، شرکت کردند. رکورد انجام آزمون ها توسط کرنومتر با دقت 01/0 ثانیه ثبت شد. یافته‌ها: پس از جمع آوری اطلاعات و بررسی نحوه توزیع داده ها از ضریب همبستگی پیرسون برای بررسی رابطه و از t دو گروه مستقل برای تعیین تفاوت بین متغیرهای تحقیق استفاده شد. سطح معنی داری کوچکتر از 01/0 برای آزمون فرضیه ها در نظر گرفته شد. نهایتاً مشاهده شد که تفاوت معنی داری بین رکورد اجرای آزمون های عملکردی جهش به شکل هشت لاتین و جهش جانبی در دو گروه آسیب دیده و سالم وجود دارد. بحث و نتیجه‌گیری: ورزشکاران دارای سابقه اسپرین مچ پا نسبت به ورزشکاران سالم دارای نقص های فانکشنال در حرکات ورزشی می باشند. همچنین با توجه به اسپرین های مکرری که در مچ پای ورزشکاران به وجود آمده و باعث افت عملکرد آنان می شود، می توان ازآزمون های عملکردی جهش به شکل هشت لاتین و جهش جانبی با صرف هزینه اقتصادی و زمانی کمی به منظور شناسایی نقص های فانکشنال و اهدافی چون پیشگیری، درمان و توانبخشی آسیب اسپرین مچ پا استفاده نمود. بنابراین مهمترین کاربرد این تحقیق: پیشگیری از وقوع اسپرین های بعدی به وسیله شناسایی افراد مستعد آسیب و ارائه راهکار های پیشگیرانه تحقق می یابد.

Title: بررسی سلامت روان بیماران نیازمند آنژیوگرافی عروق کرونر و ارتباط آن با سوء مصرف مواد

http://yafte.lums.ac.ir/article-1-69-fa.html

مقدمه: بیماری های عروق کرونر یکی از علل عمده مرگ و میر بوده که با اختلالات روانی همراهی دارند. مطالعه حاضر یک مطالعه توصیفی تحلیلی است که با هدف بررسی سلامت روان بیماران نیازمند آنژیوگرافی عروق کرونر و ارتباط آن با سوء مصرف مواد انجام گرفته است. مواد و روش‌ها: نمونه گیری به روش سرشماری بوده و جهت جمع آوری اطلاعات از پرسشنامه استاندارد GHQ و مصاحبه بالینی استفاده شده است. تمام بیماران نیازمند آنژیوگرافی پرسشنامه استاندارد 28 سوالی سلامت عمومی را تکمیل نمودند. پس از محاسبه نمره پرسشنامه تمامی بیمارانی که نمره آنها بیش از 28 بود بر اساس ملاکهای تشخیصی DSM IV- TR توسط روان پزشک مورد مصاحبه بالینی قرار گرفتند. بحث و نتیجه‌گیری: از آنجائی که بیماریهای قلبی به ویژه بیماریهای عروق کرونری اثرات منفی بر سلامت روان بیماران بر جا می گذارند، نیاز است توجهات روانی بیشتری به این بیماران مبذول گردد و راههای کنترل و مدیریت استرس به آنها آموزش داده شود. همچنین می بایست کلیه بیماران قبل از انجام پروسیجرهای اضطراب آور نظیر آنژیوگرافی تحت مشاوره و معاینه روانپزشک قرار گیرند. یافته‌ها: یافته ها نشان داد از 200 نمونه ای که در این پژوهش مورد بررسی قرار گرفتند، 5/49% از لحاظ روانی بیمار بوده و در گروه مورد بررسی به ویژه از لحاظ علائم جسمی و اختلالات اضطرابی و خواب دچار مشکل بودند. در حالیکه از نظر کارکرد اجتماعی و اختلالات افسردگی شدید سالم شناخته شدند. همچنین سوء مصرف مواد به عنوان عامل موثر در نبود سلامت روان این افراد شناخته شد. ثابت گردید که سوء مصرف مواد بخت ابتلا به بیماری روانی را در آنها بالا تر می برد و افراد دارای سوء مصرف مواد 7 برابر بیشتر از آنها که سوء مصرف مواد ندارند، شانس ابتلا به بیماری روانی را دارا هستند.

Title: بررسی علل شکست تغذیه انحصاری با شیر مادر در 6 ماه اول زندگی شیرخواران در مراجعین به مراکز بهداشتی – درمانی شهری خرم آباد درسال 1385

http://yafte.lums.ac.ir/article-1-71-fa.html

مقدمه: شواهد زیادی از فوائد شیر مادر برای سلامت نوزاد و مادر وجود دارد. در بعضی مناطق جهان میزان تغذیه انحصاری با شیر مادر در 6 ماه اول زندگی شیرخوار کاهش یافته است. همچنین مهمترین فاکتورهای مربوط با کاهش میزان تغذیه انحصاری با شیر مادر در 6 ماه اول زندگی عوامل اقتصادی - اجتماعی مانند سن مادر، وضعیت اشتغال مادر، سطح سواد مادرو فاصله بین شیرخوار از فرزند قبلی وبعدی است. مواد و روش‌ها: این مطالعه از نوع مقطعی – توصیفی تحلیلی بود. جامعه مورد مطالعه مادران دارای کودک 6 ماهه تا یکساله بودند. حجم نمونه 340 نفر بود. متغیرها شامل سن، شغل مادر، رتبه زایمان، جنس کودک،نوع زایمان تحصیلات مادر، علت تغذیه غیر انحصاری،فاصله سنی کودک فعلی با قبلی، وضعیت تغذیه انحصاری با شیر مادرو مدت تغذیه انحصاری به ماه بود. داده ها پس از جمع آوری با نرم افزار spss جهت آنالیز آماری قرار گرفت. در این تحقیق همچنین از آزمون های مانند آزمون کای- دو، آزمون دقیق فیشر، آزمون من ویتنی، آزمون کروسکال والیس و ضریب همبستگی پیرسون استفاده شد. یافته‌ها: از 340 نمونه، 151 نفر زایمان به طریق سزازین داشتند. تعداد مادران شاغل 57 نفر و تعداد مادران خانه دار 283 نفر بودند.از 340 بچه 185 نفر پسر و 155 نفر دختر بودند که 10 نفر دو قلو بودند. میزان تغذیه انحصاری با شیر مادر در 6 ماه اول زندگی 1/79% بدست آمد. 9/20% شکست در تغذیه انحصاری داشتند. از میان 268 شیرخوار که تغذیه انحصاری داشتند 144 نفر پسر و 124 نفر دختر بودند. تعداد مادرانی که تحصیلات دانشگاهی داشتند 53 نفر بودند و تعداد مادرانی که تحصیلات متوسطه داشتند 145 نفر و مادرانی که بیسواد یا کم سواد بودند 142 نفر بودند. علل شکست تغذیه انحصاری عبارتند از: افت در منحنی رشد، شاغل بودن، چند قلویی و سایر علل بود. بین شغل مادر، سواد مادر، زایمان به روش سزارین و شکست تغذیه انحصاری با شیر مادر در 6 ماه اول زندگی ارتباط معنی داری وجود داشت. دلیل اصلی برای شکست تغذیه انحصاری با شیر مادر کاهش رشد فرزندان گزارش شده بود. بحث و نتیجه‌گیری: براساس یافته های بدست آمده میزان شکست تغذیه انحصاری با شیر مادر در 6 ماه اول زندگی شیرخواردر شهر خرم آباد 9/20% بود. بنابراین ما پیشنهاد میکنیم مادران شاغل حداقل 6 ماه مرخصی زایمان جهت تغذیه انحصاری بنابه توصیه سازمان بهداشت جهانی داشته باشند، محل مناسبی برای شیردهی کودکان درمحل کار فراهم شود یا ساعت کاری کاهش یابد. مادران باید با جدول رشد نرمال بچه ها وعلل کاهش رشد آنها بیشتر آشنا باشند، همچنین با یدبه مادران در مورد فوائد شیرمادر در طی حاملگی، زایمان و در مراکز بهداشتی درمانی آموزش داده شود.

Title: مقایسه اثرات منابع کربن متانل، اتانل و سوکسینات در حذف بیولوژیکی نیترات پساب

http://yafte.lums.ac.ir/article-1-72-fa.html

مقدمه: افزایش غلظت نیترات در منابع آب یک مشکل مطرح در بسیاری از نواحی دنیا می باشد. ورود ترکیبات حاوی نیتروژن به محیط می تواند مشکلاتی نظیر اتروفیکاسیون برای منابع آب و پتانسیل خطر برای سلامتی انسان بدلیل ایجاد بیماری متهموگلوبینما و سرطان را به همراه داشته باشد پس باید نیترات را از منابع آبی حذف کرد. در میان روش های توصیه شده برای حذف این عامل، حذف بیولوژیکی نیترات یک روش موثر برای حذف نیترات از پساب و آب می باشد. مواد و روش‌ها: در این تحقیق فرآیند حذف بیولوژیکی نیترات با استفاده از منابع مختلف کربن اتانل، متانل و سوکسینات در مقیاس آزمایشگاهی مورد بررسی قرار گرفته است. در این راستا، اثر تغییر نوع منبع کربن، غلظت اولیه نیترات، pH و میزان تلقیح اولیه باکتری ارزیابی شده است. یافته‌ها: نتایج این تحقیق نشان داده است که از میان منابع کربن مورد آزمایش، باکتری سودوموناس استوتزری قادر به استفاده از متانل به عنوان منبع کربن نمی باشد. با استفاده از سوکسینات این باکتری قادر به حذف کامل نیترات با غلظت 200 میلی گرم بر لیتر بر حسب نیتروژن در دمای 28 درجه سانتیگراد، 2/7= pH و میزان تلقیح اولیه CFU/ml 108× 3 می باشد. میزان نیتریت تولیدی در این فرآیند کمتر از 1 میلی گرم بر لیتر بر حسب نیتروژن بوده است. بحث و نتیجه‌گیری: باکتری سودوموناس استوتزری مورد استفاده در این مطالعه می تواند از اتانل نیز به عنوان منبع کربن در حذف بیولوژیکی نیترات استفاده نماید اما روند کاهش نیترات برای سوکسینات بهتر از اتانل بوده است.

Title: گزارش یک مورد تومور مخچه ای همانژیوبلاستوم منجربه فوت با علائم روانی رفتاری

http://yafte.lums.ac.ir/article-1-73-fa.html

مقدمه: تومورهای مغزی یکی از کشنده ترین بدخیمی هاست. این تومورها مکرراً با تظاهرات روانی و رفتاری همراه هستند که باید مورد توجه قرار گیرد. مطالعات نشان میدهد که اختلالات روانپزشکی اولیه در 47تا 94 درصد موارد با تومورهای مغزی همراهی دارند. گزارش مورد: خانمی 26 ساله مجرد با علایم عصبی روانی تحت درمان دارویی با داروهای ضد جنون و آرام بخش قرار داشت. برای سایر علائم بیمار، درمانهای علامتی نیز انجام شد اما حال نامبرده رو به وخامت گذاشت و درنهایت بعد از 6 ماه فوت کرد. در کالبد گشایی پس از مرگ، تومور مخچه ای همانژیوبلاستوم کشف شد . بحث و نتیجه‌گیری: همانژیوبلاستوم توموری خوش خیم است و بارزکسیون، بهبودی حاصل می شود. اگر در این بیمار در ابتدای امر به علایم هشداردهنده توجه بیشتری می شد و ظن بالینی نسبت به وجود ضایعه فضاگیر مغزی بعنوان عامل ایجاد کننده علایم عصبی روانی بوجود می آمد و از حداقل روشهای تشخیص تصویربرداری مثل سی تی اسکن استفاده می شود، ما امروز شاهد مرگ انسانی با عنوان بیمار روانی نبودیم .
